# 

**Published:** 1914-12-26

**Authors:** 


### CONTENTS.

PAGE

**The Medical Profession and Public Holidays...** 275

**The Local Government Board's Opportunity...** 276

**Hospital and Institutional News ...** ... ... 277

Christmas Festivities in War-Time—East Coast Hospitals and the Raid—The New Professor of Bio-Chemistry at Cambridge—Liverpool Merchants' Movable Hospital—King's College Hospital Removal Fund—The Night Attendance of Insured Patients—An Architect as Hospital Secretary—The Shortage of Medical Officers—"Dummy" Teats and Rubber Toys—Adequate Subscriptions to Hospitals—The Women's Voluntary Hospitals Brigade—The Power of the Pence—Financial Straits of York County Hospital—Another Hospital Running Away—Mr. Lloyd George and the Red Cross—The Metropolitan Hospital Sunday Fund—The St. Pancras Dispensary Scheme—French Red Cross Society—King George Hospital—Casualties Among German Army Doctors—Death of a Distinguished Belgian Scientist—A Surcharge for Assistance at Operations—St. Katharine's Hospital Again—The Hospital Saturday Fund—The New Matron at Coventry—Foreign Languages Made Easy—This Week's Drug Market—To Our Readers.

**The Advancement of the Science of Healing** 283

By the Right Hon. the VISCOUNT MIDLETON.

**Benzol in Leukæmia ...** ... ... ... ... 284

**The New Pharmacopœia** ... ... ... ... 285

Its Omissions, Additions, Changes, and Other Features.

PAGE

**Round the World** ... ... ... ... ... ... 286

Fellow-Workers and the Roll of Honour.

**Hospitals and the War** ... ... ... ... 287

The Bombardment of the Hartlepools: Work at the Cameron Hospital — Coventry: Wounded in the New Ward—Leicester: Base Hospital—Cambridge: 1st Eastern General Hospital—Christmas Arrangements at St. Bartholomew's Hospital, Rochester—Bristol—Cardiff: Territorials from the Trenches—Tunbridge Wells.

**King Edward's Hospital Fund for London** ... 289

Annual Meeting to Award Grants.

Report of the King's Fund Distribution Committee.

Grants Recommended by the Distribution Committee.

Report of the King's Fund Convalescent Homes Committee.

**The League of Mercy** ... ... ... ... 294

Annual Meeting of Presidents at St. James's Palace.

**The Editor's Letter-Box** ... ... ... ... 298

"Canvassing Will Disqualify" (?).

**Medical Appointments** ... ... ... ... 298

**The Book Market** ... ... ... ... ... 298

**The Institutional Worker** ... ... (Suppl.) 1

The Pasteurisation of Milk.

Questions for December.

Questions Invited.

The Sick and in Need.

Employment and Training

